# Androgen receptor CAG repeat polymorphism is not associated with insulin resistance and diabetes among South Asian males

**DOI:** 10.1186/s13104-017-3035-5

**Published:** 2017-12-04

**Authors:** Lasantha S. Malavige, Surani Jayawickrama, Priyanga Ranasinghe, Jonathan C. Levy

**Affiliations:** 1 Nuffield Department of Clinical Medicine, University of Oxford, Oxford, OX3 7LJ UK; 2 Oxford Radcliffe Trust, Oxford Centre for Diabetes, Endocrinology and Metabolism, Oxford, UK; 3 Ministry of Health Care and Nutrition, Colombo, Sri Lanka; 4 Department of Pharmacology, Faculty of Medicine, University of Colombo, Colombo, Sri Lanka

**Keywords:** Androgen receptor, CAG repeats, Polymorphism, Diabetes, South Asian

## Abstract

**Objective:**

To study relationship between androgen receptor (AR) CAG repeat polymorphism, insulin resistance (IR), β-cell function and other clinical/biochemical parameters in ethnic South Asian adults. A case (males with diabetes), control (males without diabetes) study, was conducted and 110 males were invited. Anthropometry, blood pressure and biochemical parameters (fasting Insulin, blood sugar, HbA1c and lipid profile) were measured. IR and β-cell function was calculated. A multiple-linear-regression analysis was performed, using number of AR CAG repeats as the continuous dependent variable.

**Results:**

Sample size was 100 (response rate—90.9%, cases—53). Mean age was 49.6 ± 10.7 years. CAG repeat length did not show any significant correlation with IR or β-cell function. In all males there was a significant correlation between number of AR CAG repeats and systolic blood pressure (r = 0.25; p = 0.016), diastolic blood pressure (r = 0.21; p = 0.045), total cholesterol (r = − 0.22; p = 0.037) and low-density lipoprotein cholesterol (r = − 0.22; p = 0.037). Only total cholesterol (β = − 4.41; p < 0.001) and estrogen (β = 2.25; p = 0.03) were significantly associated with number of AR CAG repeats in regression analysis. In conclusion, AR CAG repeat length did not show any significant correlation with IR or β-cell function. Positive association of AR CAG with systolic and diastolic blood pressure and negative association of AR CAG with total and low-density lipoprotein cholesterol deserves further attention.

## Introduction

Males of South Asian origin, including Sri Lankans are at an increased risk of developing insulin resistance (IR) and type-2 diabetes (T2DM), in comparison to European males [1]. Central obesity significantly contributes to the pathogenesis of IR through a variety of mechanisms [2]. However, even in the absence of central obesity South Asian men have shown to have an increased IR, compared to European males [3]. There has been evidence to suggest that hypoandrogenism is associated with T2DM, metabolic syndrome and central obesity in males [4–6]. Heald et al. [7] reported significantly lower testosterone levels in a small group of South Asian males, compared to Europid and Afro-Caribbean males. It is possible that this lower testosterone levels or testosterone activity in South Asian males are associated with the higher prevalence of IR and T2DM.

Activity of testosterone is predominantly related to the activation of the androgen receptor (AR). Exon 1 of the AR gene contains a polymorphic CAG repeat sequence which encodes a variable length poly-glutamine stretch (AR CAG) [8]. The length of the CAG repeats vary between 12 and 30 [8, 9]. The number of CAG repeats in the AR has also shown to be associated with body fat content, leptin and insulin [10]. Ethnic variation in CAG repeats in the AR has been reported [11]. However, to date this has not been studied in South Asian males. It could be possible that the higher body fat content, IR and T2DM in South Asian males are associated with higher prevalence of the AR polymorphism. The present study aims to study the relationship between AR polymorphism, IR, β-cell function and other clinical/biochemical parameters in a cohort of ethnic South Asian adults with and without diabetes.

## Main text

### Methods

#### Study population and sampling

The study was conducted as a case–control study. One hundred and ten males between 21 and 65 years of age were invited for the study from the National Hospital of Sri Lanka (NHSL), which included an equal proportion of males with diabetes mellitus (cases) and males without diabetes (controls). Sample size to determine a difference of AR CAG repeats of 1 (SD 1.75) between the cases and controls (80% power and 95% confidence interval), with a non-response rate of 10% was 108. Hence, a total of 110 subjects (55 in each study group) were invited for the study. Those on anti-testosterone treatment, those who have undergone prostatic surgery or pelvic irradiation or those who are diagnosed with klinefelters syndrome were excluded. Ethical approval for the study was obtained from the Ethics Review Committee (ERC), Faculty of Medicine, University of Colombo. Informed written consent was obtained from all research participants.

#### Study instruments and data collection

A structured interviewer-administered questionnaire was used for data collection. The questionnaire evaluated socio-demographic and clinical details such as age, diabetes status and presence of other chronic diseases. Height, weight, waist circumference, hip circumference and blood pressure were measured according to standard methods. Following hormonal studies were performed by a chemiluminescent immunoassay (CLIA) using commercially available kits; total testosterone, sex hormone binding globulin (SHBG), luteinising hormone (LH), follicular stimulating hormone (FSH), estrogen (E2) and fasting insulin. In addition total cholesterol, high-density lipoprotein (HDL), low-density lipoprotein (LDL), triacylglyceride (TAG), serum albumin, fasting blood sugar (FBS) and HbA_1_C was also evaluated in all research participants. IR (HOMA-IR) and β-cell function (HOMA-β) was calculated based on the Homeostatic model assessment (HOMA) method.

#### Genetic investigations for AR polymorphism

DNA was extracted from peripheral lymphocytes in whole blood. A region in the exon 1 of the Androgen receptor gene present in the X chromosome was amplified by PCR. The PCR fragment was purified and sequenced at Macrogen Inc Korea. The sequence obtained was visualized in Bioedit sequence editor and the alignment was processed using the Mega 4 software with a known AR gene sequence obtained from the NCBI database as a reference. The final sequence was again blasted against the sequences that were reported in the Genebank in order to clarify the amplified nucleotide sequence and count the CAG repeats.

#### Statistical analysis

Data were analyzed using SPSS version 17.0 (SPSSInc., Chicago, IL, USA). A multiple linear-regression analysis was performed in all patients with number of AR CAG repeats as the continuous dependent variable and total cholesterol, LDL cholesterol, systolic blood pressure, diastolic blood pressure, IR, free testosterone, estrogen and diabetes status as the continuous or dichotomous independent variables and confounding factors. In all analyses a p value ≤ 0.05 was considered statistically significant.

### Results

Sample size was 100 (response rate—90.9%). There were 53 patients with diabetes (cases, response rate—96.4%) and 47 patients without diabetes (controls, response rate—85.4%). Mean age (± SD) was 49.6 ± 10.7 years (cases—52.2 ± 10.8 years and controls—47.3 ± 10.2 years). The clinical and biochemical characteristics of the cohort are summarized in Table 1. Except for age, BMI, FBS, HbA1c, SHBG, FSH, LH, Total cholesterol and LDL cholesterol, all other clinical and biochemical characteristics were not significantly different between patients with and without diabetes. The calculated mean HOMA-IR (± SD) in the patients with diabetes was 1.5 ± 0.7, while in patients without diabetes it was 1.0 ± 0.5 (p < 0.01). Mean β-cell function (HOMA-β %) (± SD) in patients with and without diabetes was 83.1 ± 75.3 and 121.4 ± 62.4 respectively (p = 0.02).

**Table 1:** Clinical and biochemical features of the cases and controls

	Cases (diabetics)	Controls (non-diabetic)	p value
Age (years)	52.2 ± 10.8	47.3 ± 10.2	0.02
BMI (kg/m^2^)	24.2 ± 3.1	25.4 ± 3.2	0.05
Waist circumference (cm)	90.2 ± 8.0	92.0 ± 8.9	0.28
Hip circumference (cm)	93.5 ± 6.9	95.0 ± 7.5	0.30
Systolic blood pressure (mmHg)	123.2 ± 16.9	123.2 ± 13.9	0.98
Diastolic blood pressure (mmHg)	81.5 ± 9.1	83.8 ± 10.6	0.24
Hypertension	24 (51.1%)	19 (35.8%)	0.16
Ischaemic heart disease	18 (38.3%)	14 (26.4%)	0.28
Stroke	3 (6.4%)	5 (9.4%)	0.72
Cirrhosis	1 (2.1%)	1 (1.9%)	0.92
Chronic kidney disease	4 (8.5%)	2 (3.8%)	0.42
FBS (mg/dL)	141.3 ± 75.2	83.2 ± 25.7	< 0.001
HbA1C (%)	8.7 ± 2.1	5.9 ± 0.89	< 0.001
Total Testosterone (ng/dL)	554.3 ± 218.6	569.5 ± 257.4	0.75
Free testosterone (ng/dL)	9.9 ± 7.1	12.2 ± 8.9	0.15
Bio-available testosterone (ng/dL)	202.2 ± 121.5	239.6 ± 148.3	0.17
SHBG (nmol/L)	59.9 ± 28.5	45.6 ± 23.8	0.007
FSH (mIU/mL)	9.8 ± 10.8	5.6 ± 6.1	0.02
LH (mIU/mL)	7.6 ± 4.3	4.7 ± 3.4	< 0.001
Total cholesterol (mg/dL)	209.6 ± 72.4	239.6 ± 69.9	0.04
LDL (mg/dL)	133.2 ± 61.4	162.5 ± 61.8	0.02
HDL (mg/dL)	47.7 ± 13.8	45.9 ± 14.7	0.54
Triglycerides (mg/dL)	143.0 ± 81.2	155.6 ± 76.7	0.42

* Continuous variables are presented as mean ± SD and frequencies as n (%)

**Fig. 1:**
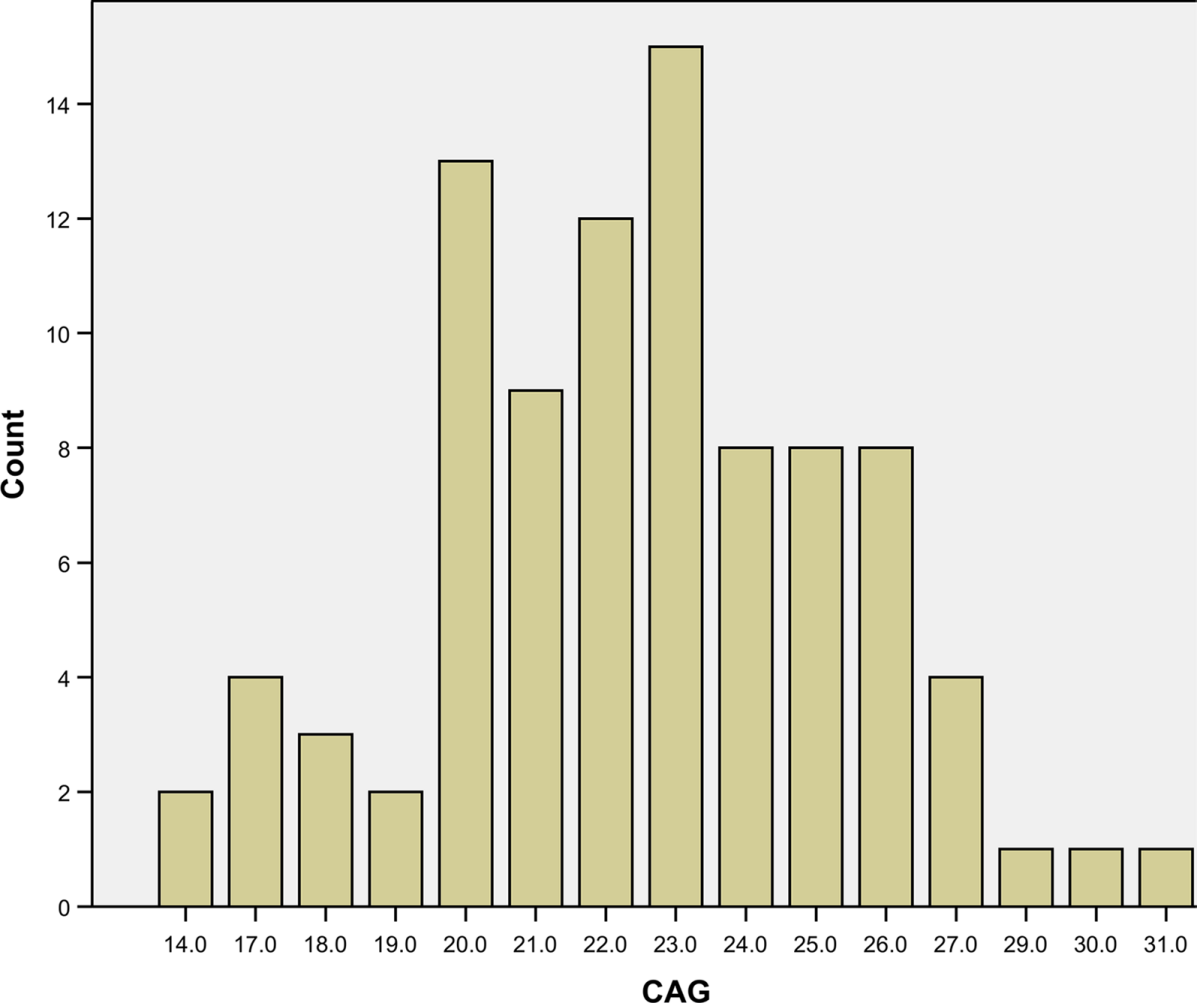
Distribution of the androgen receptor CAG repeat lengths in the entire study population (both cases and controls)

The mean CAG repeat length (± SD) of the AR in the study cohort was 22.4 ± 3.1 ranging from 14 to 31 as shown in Fig. 1. The mean AR CAG repeat length (± SD) in patients with and without diabetes was 22.3 ± 3.2 and 22.6 ± 3.0 respectively (p = 0.70). The AR CAG repeat length did not show any significant correlation with IR in all males (*r* = 0.05; p = 0.68), cases (*r* = 0.15; p = 0.46) or controls (*r* = 0.01; p = 0.93). Similarly no significant correlation was observed between the AR CAG repeat length and β-cell function (HOMA-β %) in all males (*r* = − 0.04; p = 0.72), patients with (*r* = − 0.09; p = 0.62) and without diabetes (*r* = − 0.02; p = 0.88). The mean number of CAG repeats in the AR gene did not show any significant difference with the presence of hypertension, ischaemic heart disease, stroke, cirrhosis and chronic kidney disease. Similarly the mean number of CAG repeats in the AR gene did not show any significant difference with the presence of metabolic syndrome defined according to the NCEP-ATPIII criteria.

In the study cohort there was no significant correlation between the number of AR CAG repeats and BMI (*r* = 0.09; p = 0.41), waist circumference (*r* = 0.05; p = 0.61) and hip circumference (*r* = 0.004; p = 0.97). A similar observation was noted independently in both males with and without diabetes (data not shown). In all males there was a significant positive correlation between the number of AR CAG repeats with the systolic (*r* = 0.25; p = 0.016) and diastolic blood pressure (*r* = 0.21; p = 0.045). However, this association was not observed independently in males with and without diabetes.

In the study cohort the number of AR CAG repeats did not shown any significant correlation with FBS (*r* = 0.03; p = 0.75), HbA1c (*r* = − 0.07; p = 0.53), serum insulin (*r* = 0.05; p = 0.65), triglycerides (*r* = − 0.09; p = 0.37), HDL cholesterol (*r* = − 0.05; p = 0.66), total testosterone (*r* = − 0.06; p = 0.57), free testosterone (*r* = 0.10; p = 0.34), bio-available testosterone (*r* = 0.02; p = 0.81), albumin (*r* = 0.05; p = 0.66), SHBG (*r* = − 0.11; p = 0.29), FSH (*r* = − 0.02; p = 0.89) LH (*r* = 0.05; p = 0.67) and Estrogen (*r* = 0.25; p = 0.08). A similar finding was seen in males with and without diabetes independently (data not shown). In all males a significant negative correlation was observed between the number of AR CAG repeats and total cholesterol (*r* = − 0.22; p = 0.037) and LDL cholesterol (*r* = − 0.22; p = 0.037). A higher negative correlation between the number of AR CAG repeats and total cholesterol (*r* = − 0.44; p = 0.003) and LDL cholesterol (*r* = − 0.45; p = 0.001) was observed in males with diabetes. However, no significant correlation was observed in males without diabetes. The backward regression model demonstrated statistical significance, the Cox & Snell R-Square and Nagelkerke R Square values were 0.379 and 0.339 respectively. The final results indicated that only total cholesterol (β = − 4.41; p < 0.001) and estrogen (β = 2.25; p = 0.03) were significantly associated with the number of AR CAG repeats.

### Discussion

In the present study we explored the relationship between AR CAG repeat length, IR, diabetes status and other clinical/biochemical parameters in a cohort of ethnic South Asian males. The mean CAG repeat length (± SD) of the AR in the present study cohort was 22.4 ± 3.1 (range 14–31), which is comparable to results observed previously in white Caucasian males (22.0 ± 3.2 [range 17–32]) [12]. A large cohort study among 2878 males from eight different European countries also demonstrated a similar result (mean 22.1 ± 3.1) [8]. Rajan et al. [13] evaluated the AR CAG repeat length in a group of Indian males residing in USA and demonstrated that their mean number of AR CAG repeats (22.3 ± 3.7) were similar to that of white Caucasians (22.4 ± 3.1) taken as age-matched controls. The result of the present study also supports the above findings. It is evident from these studies that the mean number of AR CAG repeats does not differ significantly between the white Caucasian and South Asian males.

We were unable to demonstrate any significant relationship between the number of AR CAG repeats and the presence of diabetes, hypertension, ischaemic heart disease, stroke, cirrhosis and chronic kidney disease. Similarly the AR CAG repeat length did not show any significant correlation with IR, β-cell function, FBS and HbA1c. In patients with diabetes IR and β-cell function is likely to be modified by treatment and progression of the disease. Furthermore, it is not unexpected that AR CAG had no relationship with FBS and HbA1C in our population, because patients with diabetes are on treatment with diet, oral hypoglycaemics and insulin with the aim of meeting diabetes treatment targets and in patients without diabetes the parameters are remaining within the normal range. However, previous studies have demonstrated a significant relationship between AR CAG repeat polymorphism and body fat mass, serum leptin, serum insulin and IR among healthy males [12, 14]. Hence, the lack of association observed in the healthy cohort in the present study requires further investigation. We also did not observe any significant relationship between AR CAG repeat length and serum testosterone (free/bio-available), SHBG, FSH and LH. Some studies have found a positive correlation between testosterone levels and AR CAG repeat length in healthy men [15]. A finding which has not been supported by studies in other male populations, including the present study [16, 17]. This could be due to the fact that testosterone can have actions independent of the AR, with newer evidence demonstrating its action in a non-genomic way at the cell surface [18].

The positive association of AR CAG with systolic and diastolic blood pressure deserves further attention. The fact that these findings were not independently observed in those with and without diabetes may suggest a false positive result. However, since this has previously been observed in adolescents, hypo-gonadal males and in males with diabetes, this association deserves further study, to establish or refute any relationship and to identify the aetio-pathological mechanisms [10, 19, 20]. We also observed a significant negative correlation between the number of AR CAG repeats, total cholesterol and LDL cholesterol in males with diabetes. Previous studies have demonstrated that a higher AR CAG repeat length is associated with an increase in LDL cholesterol and triglyceride, while showing a negative correlation with HDL and total cholesterol [21]. It is likely that the lipid profile was modified by lipid lowering drugs (57.4%) in the diabetic cohort, resulting in a false association. This is supported by the fact that no significant correlation was observed independently in males without diabetes, a finding also supported by evidence from previous studies [22].

## Limitations

There are several limitations which needs to be appreciated. It is a single-centre study on a limited number of patients. However, the study findings can serve as cornerstones for further studies involving a larger number of patients. Furthermore, most of the participants in the diabetes cohort were treated with medications that could affect BMI, waist, HbA1C and blood pressure levels, which could distort associations between AR CAG repeat length and other variables. The AR CAG distribution in our population is similar in comparison to other populations both Asian and Caucasian, implying representativeness of the sample. It is also important to note that this cross-sectional study can only describe relationships between AR CAG and other variables and does not provide evidence of causal effects.

## Abbreviations

AR: androgen receptor
BMI: body mass index
FBS: fasting blood sugar
FSH: follicular stimulating hormone
HbA1c: glycosylated haemoglobin
HDL: high density lipoprotein
IR: insulin resistance
LDL: low density lipoprotein
LH: leutinising hormone
SHBG: sex hormone binding globulin
USA: United States of America
